# Prevalence of Common Ocular Morbidities in Adult Population of Aligarh

**DOI:** 10.4103/0970-0218.55283

**Published:** 2009-07

**Authors:** Inaamul Haq, Zulfia Khan, Najam Khalique, Ali Amir, Fatima A Jilani, Meena Zaidi

**Affiliations:** Department of Community Medicine & Institute of Ophthalmology, Jawaharlal Nehru Medical College, Aligarh Muslim University, Aligarh, Uttar Pradesh, India

**Keywords:** Adult, ocular morbidities, prevalence

## Abstract

**Aims and Objectives:**

To determine the prevalence of common ocular morbidities (cataract, refractive errors, glaucoma, and corneal opacities) and their demographic and sociocultural correlates.

**Settings and Design:**

The present cross-sectional study was conducted in the field practice areas of the Department of Community Medicine, JNMC, AMU, Aligarh, for a period of one year, from September 2005 to August 2006.

**Materials and Methods:**

Systematic random sampling was done to select the required sample size. All adults aged 20 years and above in the selected households were interviewed and screened using a 6/9 illiterate ‘E’ chart. Those who could not read the ‘E’ chart were referred to the respective health training center for a complete eye examination by an ophthalmologist.

**Statistical Analysis:**

Chi- square test.

**Results:**

The prevalence of visual impairment, low vision, and blindness, based on presenting visual acuity was 13.0, 7.8, and 5.3%, respectively. The prevalence of cataract was 21.7%. Bilateral cataract was present in 16.9% of the population. Cataract was significantly associated with age, education, and fuel use. The prevalence of myopia, hypermetropia, and astigmatism was 11.5, 9.8, and 3.7%, respectively. Glaucoma was diagnosed in six patients, giving a prevalence rate of 0.9%. All the six patients of glaucoma were aged above 40 years. The prevalence of corneal opacity was 4.2%.

**Conclusion:**

There is a high prevalence of treatable or preventable morbidities such as cataract, refractive errors, and corneal opacity.

## Introduction

About 161 million people are visually disabled in the world today, and the number is steadily increasing because of population growth and aging.[1] Blindness, with its social and economic consequences, represents a significant public health problem in many parts of the world.[2]

Cataract is the most common easily correctable cause of blindness in the developing regions of the world.[3] In India alone 3.8 million people become blind from cataract each year.[4] In many parts of the world refractive errors would become the second largest cause of treatable blindness after cataract, if the blindness were defined on the basis of 'presenting' distance visual acuity.[5] Glaucoma is now the second leading cause of blindness globally, after cataract.[1] The epidemiology of corneal blindness is complicated and encompasses a wide variety of infectious and inflammatory eye diseases that cause corneal scarring, which ultimately leads to functional blindness. Trachoma, ocular trauma, and corneal ulceration are significant causes of corneal blindness, which are often underreported, but may be responsible for millions of new cases of monocular blindness every year.[6]

Considering the complicated epidemiology of visual impairment and the wide variety of factors involved, region specific intervention strategies are required for every community. Therefore, providing appropriate data is one of the first steps in these communities. Various studies estimating the burden of visual impairment and blindness in the elderly have been conducted in various parts of the country in the past. However, there has been a lack of appropriate community-based data on the prevalence of ocular morbidities in adults. Thus, in view of the importance of the problem of ocular morbidities and the lack of appropriate community-based data on their prevalence in adults, especially in and around Aligarh, the present study was undertaken with the following aims and objectives:

1. To determine the prevalence of common ocular morbidities (cataract, refractive errors, glaucoma, and corneal opacities) in the study population.
2. To determine the sociocultural and demographic factors in relation to the ocular morbidities.

## Materials and Methods

The present cross-sectional study entitled, “Prevalence of Common Ocular Morbidities in an Adult Population of Aligarh,” was conducted for a period of one year, from September 2005 to August 2006, in the field practice areas of the Urban and Rural Health Training Centers of the Department of Community Medicine, Jawaharlal Nehru Medical College, Aligarh Muslim University, Aligarh. The Urban Health Training Center caters to a predominantly peri-urban area situated on the outskirts of the city. The Rural Health Training Center is situated at Jawan, a Block headquarter village, about 15 kms from the city. The Training Centers cater to a total of 3324 households with a total registered adult population of 13121. The adult population (20 years and above) in households registered with the Urban and Rural Health Training Centers of the Department of Community Medicine was selected for the present study. A Systematic Random Sampling design was used to select a sample size of 700 adults; based on a 7% anticipated prevalence of glaucoma,[7] for a 95% confidence interval, a precision of 0.02 and a nonresponse rate of 10%.

All adults aged 20 years and above residing in households registered with the Urban or Rural Health Training Center and consenting for interview and examination were included in the study. A resident was defined as, “a person residing in the household for at least six months prior to the study”. People refusing interview or examination were excluded from the study.

A baseline data of the registered households was collected from the Urban and Rural Health Training Centers. The selected households were visited by the investigator. All adults in the households, aged 20 years and above, were explained the purpose of the visit and the study, and an oral consent was taken for interview and examination. The selected persons were interviewed according to a pre-designed and pre-tested proforma. Details about the socio-demographic characteristics were obtained. Modified Prasad's Classification was used to calculate the social class of an individual.[8] Details about the presence of any complaints related to eye diseases, past history and family history were noted. All persons were screened by a 6/9 illiterate ‘E’ chart. A torchlight examination of the globe, eyebrows, eyelashes, eyelids, conjunctiva, sclera, cornea, pupil, iris, and lens was made in a dimly lit room of the house. Digital tonometry was done and the results noted. Subjects with any one of the conditions mentioned below were called to the respective health training centers for a detailed ocular examination.

1. ‘Cannot read’ the 6/9 ‘E’ chart with either eye.
2. Presence of symptom(s) and/or sign(s) of ocular disease.
3. Family history of glaucoma.

Detailed ocular examination of the invited persons at the respective health training centers included a ‘presenting’ and ‘best corrected’ visual acuity using Snellens chart, retinoscopy using a streak retinoscope, measurement of intraocular pressure using “Keeler Pulsair 3000 Easy Eye” tonometer (Keeler Instruments, Inc., Broomhall, Pennsylvania), and direct ophthalmoscopy. The following definitions were used for the study:

Visual impairment: A ‘presenting’ visual acuity of < 6/18 in the better eye.[9]

Low vision: A ‘presenting’ visual acuity of < 6/18, but ≥ 6/60 in the better eye.[9]

Blindness: A ‘presenting’ visual acuity of < 6/60 in the better eye.[9]

Cataract: Lens opacity accompanied by or capable of causing some level of visual loss.[10]

Corneal opacity: Loss of normal transparency of the cornea.

Glaucoma: An intraocular pressure of more than 21 mm Hg accompanied by a horizontal or vertical cup-disc ratio difference ≥ 0.6 or a horizontal or vertical cup-disc ratio difference ≥0.2.[11]

Myopia: Myopia was defined as a spherical equivalent less than – 0.50 diopter sphere (DS).[12]

Hypermetropia: Hypermetropia was defined as a spherical equivalent greater than + 0.50 DS.[12]

Astigmatism: Astigmatism was defined as a cylindrical error more than 0.50 diopter cylinder (DC) in any axis.[12]

Data entry and statistical analysis: Data entry and statistical analysis were done by using SPSS 10.0. Univariate analysis was done with the help of the chi-square test, computed using SPSS 10.0. Mantel-Haenszel chi-square test (χ^2^_MH_) was used to control any possible confounding variable, wherever necessary. A *P* value of less than 0.05 was taken as significant.

## Results

A total of 700 persons aged 20 years and above were contacted, of whom 55 (7.9%) did not give consent or did not turn up for examination and were excluded from the study. Of the remaining 645 persons, 226 were called to the respective health training center for further examination.

The socio-demographic characteristics of the study population are shown in Table 1. Majority of the participants (31.9%) belonged to the 20-29 years age group, while the least contribution (6.0%) was by those aged 70 years and above. At least three visits were paid to the chosen households, to interview and examine all the adults in the household. A family history of glaucoma was present in 10 persons.

**Table 1:** Distribution of study population according to socio-demographic characteristics

Socio-demographic characteristics	Residence		Total (n = 645)
		
	Urban (n = 321)	Rural (n = 324)	
Age (years)			
20–29	118 (36.8)	88 (27.2)	206 (31.9)
30–39	76 (23.7)	56 (23.7)	132 (20.5)
40–49	56 (17.4)	50 (15.4)	106 (16.4)
50–59	34 (10.6)	57 (17.6)	91 (14.1)
60–69	22 (6.9)	49 (15.1)	71 (11.0)
70 and above	15 (4.7)	24 (7.4)	39 (6.0)
Gender			
Male	134 (41.7)	129 (39.8)	263 (40.8)
Female	187 (58.3)	195 (60.2)	382 (59.2)
Religion			
Muslim	238 (74.1)	40 (12.3)	278 (43.1)
Hindu	70 (21.8)	284 (87.7)	354 (54.9)
Christian	13 (4.0)	0 (0.0)	13 (2.0)
Social class			
Class I	0 (0.0)	1 (0.3)	1 (0.2)
Class II	3 (0.9)	1 (0.3)	4 (0.6)
Class III	8 (2.5)	8 (2.5)	16 (2.5)
Class IV	45 (14.0)	25 (7.7)	70 (10.9)
Class V	265 (82.6)	289 (89.2)	554 (85.9)
Education			
Illiterate	142 (44.2)	178 (54.9)	320 (49.6)
Just literate	45 (14.0)	32 (9.9)	77 (11.9)
Primary school	34 (10.6)	33 (10.2)	67 (10.4)
Middle school			
High school	24 (7.5)	32 (9.9)	56 (8.7)
Intermediate	39 (12.1)	19 (5.9)	58 (9.0)
Graduate	15 (4.7)	18 (5.6)	33 (5.1)
Postgraduate or	18 (5.6)	6 (1.9)	24 (3.7)
Professional	4 (1.2)	6 (1.9)	10 (1.6)
Fuel used			
LPgas	101 (31.5)	28 (8.6)	129 (20.0)
Wood, coal, cow dung Both	145 (45.2)	224 (69.1)	369 (57.2)
	75 (23.4)	72 (22.2)	147 (22.8)

Visual acuity: The distribution of the study population according to ‘presenting’ and ‘best corrected’ visual acuity in the better eye is shown in Table 2. The overall prevalence of visual impairment, low vision, and blindness based on ‘presenting’ visual acuity was thus 13.0, 7.8, and 5.3%, respectively. Based on ‘best corrected’ visual acuity, the corresponding figures dropped to 7.4, 3.9, and 3.6%, respectively.

**Table 2:** Distribution of study population according to presenting and best corrected visual acuity in the better eye

Category of visual impairment	Visual acuity in the better eye		Total number (%)
No visual impairment	≥ 6/18	Presenting	561 (87.0)
		Best corrected	597 (92.6)
Visual impairment			
Low vision			
1	<6/18 - ≥6/60	Presenting	50 (7.8)
		Best corrected	25 (3.9)
2	<6/60 - ≥3/60	Presenting	16 (2.5)
		Best corrected	10 (1.6)
Blindness			
3	<3/60 - ≥ 1/60 (finger counting at 1 meter)	Presenting	16 (2.5)
		Best corrected	11 (1.7)
4	<1/60 (finger counting at 1 meter) – Light perception	Presenting	2 (0.3)
		Best corrected	2 (0.3)
Total		Presenting	645 (100.0)

Cataract: The overall prevalence of cataract (excluding aphakia) was found to be 21.7%. Bilateral cataract was present in 16.9% of the population, while another 4.8% had unilateral cataract. If aphakics were included, the prevalence of cataract would rise to 23.6% [Table 3].

**Table 3:** Distribution of ocular morbidities in the study population

	Urban (n = 321)	Rural (n = 324)	Total (n = 645)	
Cataract			
Right eye only	9 (2.8)	8 (2.5)	17 (2.6)
Left eye only	5 (1.6)	9 (2.8)	14 (2.2)
Bilateral	38 (11.8)	71 (21.9)	109 (16.9)
No cataract	269 (83.8)	236 (72.8)	505 (78.3)
Aphakia				
Right eye only	3 (0.9)	6 (1.8)	9 (1.4)	
Left eye only	7 (2.2)	2 (0.6)	9 (1.4)	
Bilateral	4 (1.3)	8 (2.5)	12 (1.9)	
No aphakia	307 (95.6)	308 (95.1)	615 (95.3)	
Refractive errors				
Myopia	42 (13.1)	32 (9.9)	74 (11.5)	
Hypermetropia	24 (7.5)	39 (12.0)	63 (9.8)	
Astigmatism	13 (4.0)	11 (3.4)	24 (3.7)	
Absent	242 (75.4)	242 (74.7)	484 (75.0)	
Glaucoma				
Present	3 (0.9)	3 (0.9)	6 (0.9)	
Absent	318 (91.9)	321 (91.9)	639 (91.9)	
Corneal opacity				
Right eye only	6 (1.9)	8 (2.5)	14 (0.5)	
Left eye only	5 (1.6)	2 (0.6)	7 (1.1)	
Bilateral	1 (0.3)	5 (1.5)	6 (0.9)	
Absent	309 (96.3)	309 (95.4)	618 (95.8)	
				

Cataract was significantly associated with age, education, and fuel use [Table 4]. The prevalence of cataract increased from 0.5% in the age group of 20 – 29 years to 82.1% in those aged 70 years and above (*P* < 0.001). The prevalence of cataract was highest in illiterates (32.8%) and decreased with increasing levels of education (*P* < 0.001). People using only solid fuels (firewood, coal, cow dung) had a significantly higher prevalence of cataract (24.9%) than those using only LPG (14.0%) (*P* = 0.031). Cataract was not related to gender (*P* = 0.427), residence (χ^2^_MH_ = 0.093; *P* = 0.760) or social class (*P* = 0.812) [Table 4].

**Table 4:** Association of cataract with socio-demographic characteristics

Socio-demographic characteristics	Total (n = 645)	Number with cataract	Prevalence(%)
Age (years)			
20–29	206	1	0.5
30–39	132	4	3.0
40–49	106	6	5.7
50–59	91	40	44.0
60–69	71	57	80.3
70 and above	39	32	82.1
Total	645	140	21.7
χ^2^ = 351.164, df = 5, *P*<0.001			
Gender			
Male	263	53	20.2
Female	382	87	22.8
Total	645	140	21.7
χ^2^= 0.630, df = 1, *P* =0.427			
Residence			
Urban	321	52	16.2
Rural	324	88	27.2
Total	645	140	21.7
χ^2^_MH_ = 0.093, *P*=0.760			
Social class			
Higher class (I, II, and III)	21	5	23.8
Lower class (IV andV)	624	135	21.6
Total	645	140	21.7
χ^2^ =0.057, df = 1, *P* =0.812			
Education			
Illiterate	320	105	32.8
Just literate	77	16	20.8
Primary school	67	11	16.4
Middle school	56	2	3.6
High school	58	2	3.4
Intermediate	33	3	9.1
Graduate	24	1	4.2
Postgraduate or professional	10	0	0.0
Total	645	140	21.7
χ^2^= 56.790, df = 7, *P*<0.001			
Fuel used			
LPGas	129	18	14.0
Wood, coal, cow dung	369	92	24.9
Both	147	30	20.4
Total	645	140	21.7
χ^2^ =6.968, df = 2, *P* =0.031			

Refractive errors: Out of the 645 people examined, refractive error was present in 161 persons. The overall prevalence of refractive errors was 25.0%. Myopia was the most prevalent refractive error (11.5%), followed by hypermetropia (9.8%), and astigmatism (3.7%) [Table 3].

Myopia was significantly related to age (*P* < 0.001). Prevalence of myopia increased from 4.4% in the age group of 20 – 29 years to 23.1% in the 50 – 59 year age group, but thereafter decreased to 12.8% for those aged above 70 years. Myopia was not related to gender (*P* = 0.086), residence (*P* = 0.201), social class (*P* = 0.681) or education. The prevalence of myopia was, however, lowest in illiterates (8.1%) and increased with higher levels of education, being highest in those with 12 completed years of education (21.2%), although this relationship was not statistically significant (*P* = 0.094) [Table 5].

**Table 5:** Association of refractive errors with socio-demographic characteristics

Socio-demographic characteristics	Total (n = 645)	Myopia		Hypermetropia		Astigmatism	
				
		Number	Prevalence (%)	Number	Prevalence (%)	Number	Prevalence (%)
Age (years)							
20–29	206	9	4.4	0	0.0	0	0.0
30–39	132	11	8.3	7	5.3	4	3.0
40–49	106	20	18.9	22	20.8	6	5.7
50–59	91	21	23.1	21	23.1	4	4.4
60–69	71	8	11.3	10	14.1	5	7.0
70 and above	39	5	12.8	3	7.7	5	12.8
Total	645	74	11.5	63	9.8	24	3.7
		χ^2^ =29.361, df = 5, *P*< 0.001		χ^2^ = 59.785, df = 5, *P*< 0.001		χ^2^ = 14.826, df = 2##, *P* = 0.001	
Gender							
Male	263	37	14.1	24	9.1	8	3.0
Female	382	37	9.7	39	10.2	16	4.2
Total	645	74	11.5	63	9.8	24	3.7
		χ^2^ = 2.946, df = 1, *P* =0.086		χ^2^ = 0.208, df = 1, *P*= 0.649		χ^2^ = 0.572, df = 1, *P*= 0.450	
Residence							
Urban	321	42	13.1	24	7.5	13	4.0
Rural	324	32	9.9	39	12.0	11	3.4
Total	645	74	11.5	63	9.8	24	3.7
		χ^2^ = 1.633, df = 1, *P*=0.201		χ^2^ = 3.805, df = 1, *P*=0.051		χ^2^ = 0.193, df = 1, *P*=0.660	
Social class							
Higher class (I, II, and III)	21	3	14.3	3	14.3	2	9.5
Lower class (IV and V)	624	71	11.4	60	9.6	22	3.5
Total	645	74	11.5	63	9.8	24	3.7
		χ^2^ = 0.169, df = 1, *P* = 0.681		χ^2^ = 0.503, df = 1, *P* = 0.478		χ^2^ = 2.040, df = 1, *P* = 0.153	
Education							
Illiterate	320	26	8.1	38	11.9	12	3.8
Just literate	77	10	13.0	10	13.0	6	7.8
Primary school	67	9	13.4	4	6.0	3	4.5
Middle school	56	10	17.9	3	5.4	1	1.8
High school	58	9	15.5	2	3.4	0	0.0
Intermediate	33	7	21.2	3	9.1	1	3.0
Graduate	24	1	4.2	2	8.3	1	4.2
Postgraduate or professional	al 10	2	20.0	1	10.0	0	0.0
Total	645	74	11.5	63	9.8	24	3.7
		χ^2^ = 12.199, df = 7, *P* = 0.094		χ^2^ = 7.552, df = 7, *P*= 0.374		χ^2^ = 4.723, df = 2£, *P* = 0.094	

## For analysis, age groups 40 – 49 and above were clubbed so that not more than 20% cells had an expected count < 5.

£ For analysis, just literate and primary school categories were clubbed, and middle school and higher categories were clubbed so that no cell had an expected count < 1 and not more than 20% cells had an expected count < 5.

Hypermetropia was significantly associated with age (*P* < 0.001). The prevalence of hypermetropia increased from 5.3% in those aged 30 – 39 years to 23.1% in those aged 50 – 59 years, but decreased thereafter to 7.7% in those aged 70 years and above. Hypermetropia was not significantly associated with gender, residence, social class or education [Table 5].

The prevalence of astigmatism was found to increase significantly with age. The prevalence was 3.0% in the age group of 30 – 39 years and increased to 12.8% in those aged 70 years and above (*P* = 0.001) [Table 5].

Glaucoma: Glaucoma was diagnosed in six patients, giving a prevalence rate of 0.9% [Table 3]. All the six patients of glaucoma were aged above 40 years. The prevalence of glaucoma increased with age from 1.2% in the age group of 40 – 49 years to 6.3% in the age group of 60 – 69 years. Five of the patients were females and belonged to the lower social class.

Corneal opacity: The prevalence of corneal opacity was 4.2%. Bilateral corneal opacity was prevalent in 0.9% of the population, while another 3.3% had unilateral corneal opacity. The prevalence of corneal opacity was 3.7% in the urban area compared to 4.6% in the rural area (*P* > 0.50) [Table 3]. Ocular trauma, corneal ulcer, and trachoma were the common causes of corneal opacity. The prevalence of corneal opacity was highest (19.7%) in the age group of 60 – 69 years. In females, the prevalence was 4.5% as compared to 3.8% in males (*P* > 0.50) [Table 6].

**Table 6:** Distribution of corneal opacity in the study population by age and gender

Age (years)	Total	Number with corneal opacity	Prevalence(%)
20–29	206	1	0.5
30–39	132	0	0.0
40–49	106	2	1.9
50–59	91	8	8.8
60–69	71	14	19.7
70 and above	39	2	5.1
Total	645	27	4.2
Gender			
Male	263	10	3.8
Female	382	17	4.5
Total	645	27	4.2

χ^2^ = 0.163, *P*>0.50

## Discussion

High cataract prevalence rates have been reported from several other studies in India. In a rural area of Pondicherry, the prevalence was found to be 27.7% in those aged 30 years and above.[13] The prevalence rate for a similar age group in the present study was 31.7%. In the Aravind Comprehensive Eye Survey, the prevalence of cataract in those aged 40 years and above was found to be 47.5%.[14] This is similar to the results of the present study (44.1% in the same age group). Higher cataract prevalence rates have been reported by several other studies in India.[15,16] Lower prevalence rates have, however, been reported from Punjab[17] and Maharashtra.[18] The close association of cataract with increasing age has been well documented by studies in India[14,15,17–19] and abroad.[20–23] Age appeared to confound the results of an association between the area of residence and cataract; a Mantel-Haenszel chi-square test revealed that the area of residence was not related to cataract (*P* = 0.760, Table 4). Similar results have been reported in a study from south India.[24]

The prevalence of myopia in the present study was low as compared to that reported by Dandona *et al*. (19.39% in > 15 years)[25] and Raju *et al*. (30.68% in > 39 years).[12] Referral criteria used in the present study might have led to a lower prevalence of myopia, since only people with a visual acuity < 6/9 were referred for evaluation of refractive error status. Myopia has been found to be significantly related to age by Dandona *et al*.[25] and Raju *et al*.[12] In both these studies the prevalence increased from the age group of 20 – 29 years to the age group of 50 – 59 years, but decreased thereafter. However, myopia has been reported to decrease with increasing age by studies outside India.[26–28]

Prevalence rates of hypermetropia similar to our study have been reported by the Andhra Pradesh Eye Disease Study.[25] In this study, 9.83% of the population, aged above 15 years, was found to have hypermetropia of > + 0.50 DS. The prevalence was, however, lower than that reported by Raju *et al*.,[12] who reported a prevalence of 8.70% in people aged above 39 years compared to 18.2% calculated for a similar age group in our study (18.2%). A possible reason might be a high prevalence of cataract in the present study. Several studies in India[12,25] and abroad[27] have reported an increasing prevalence of hypermetropia up to the fifth decade and a decrease in its prevalence thereafter, conforming to the results of our study. However, significantly increased levels of hypermetropia in older age groups are reported in several studies outside India.[26,28,29]

The prevalence of astigmatism in the present study was lower than that reported by other studies in India.[12,25] Dandona *et al*. have reported a prevalence of 12.94% in people aged above 15 years.[25] The prevalence of astigmatism in people aged above 39 years has been reported to be 54.78% in a rural south Indian population.[12] The referral criteria used in the present study might have given rise to a low prevalence of astigmatism in the present study. An increasing prevalence of astigmatism with age has been reported in several studies.[12,25,29]

The prevalence of glaucoma in the present study (0.9%) was lower than that reported by several Indian studies. The prevalence of glaucoma has been found to range from 2.6% to 7.2%.[8,18,30–33]

The prevalence of corneal opacity was high in the present study population when compared to other studies. Singh *et al*. have reported a prevalence of 2.99% in people aged above 50 years.[18] Poor knowledge about ocular health coupled with poor availability and use of eye healthcare services in our study area might be a possible reason for a higher prevalence of corneal opacity.

Limitations of the study: Females were over represented in our study because males were, most of the time, out of their houses earning their livelihood. Another drawback of the study was the definition used for glaucoma, which may have led to an underestimation of the prevalence of glaucoma.

## Conclusion

The present study suggests that there is a high prevalence of cataract, refractive errors, and corneal opacity in the study population, all of which are treatable or preventable. There is, thus, a need to define the priorities for eye care services based on the current population-based data. Thus, short-term emphasis should be placed on cataract and refractive errors, and long-term emphasis should include glaucoma and corneal diseases as well. People should be educated about their causes, preventive measures, and appropriate treatment. Health education programs should target older age groups specifically and the population in general. The availability and accessibility of eye care services, particularly cataract surgery and refraction services, should be increased. Affordable eye care services should be provided in addition to making these services available and accessible.
